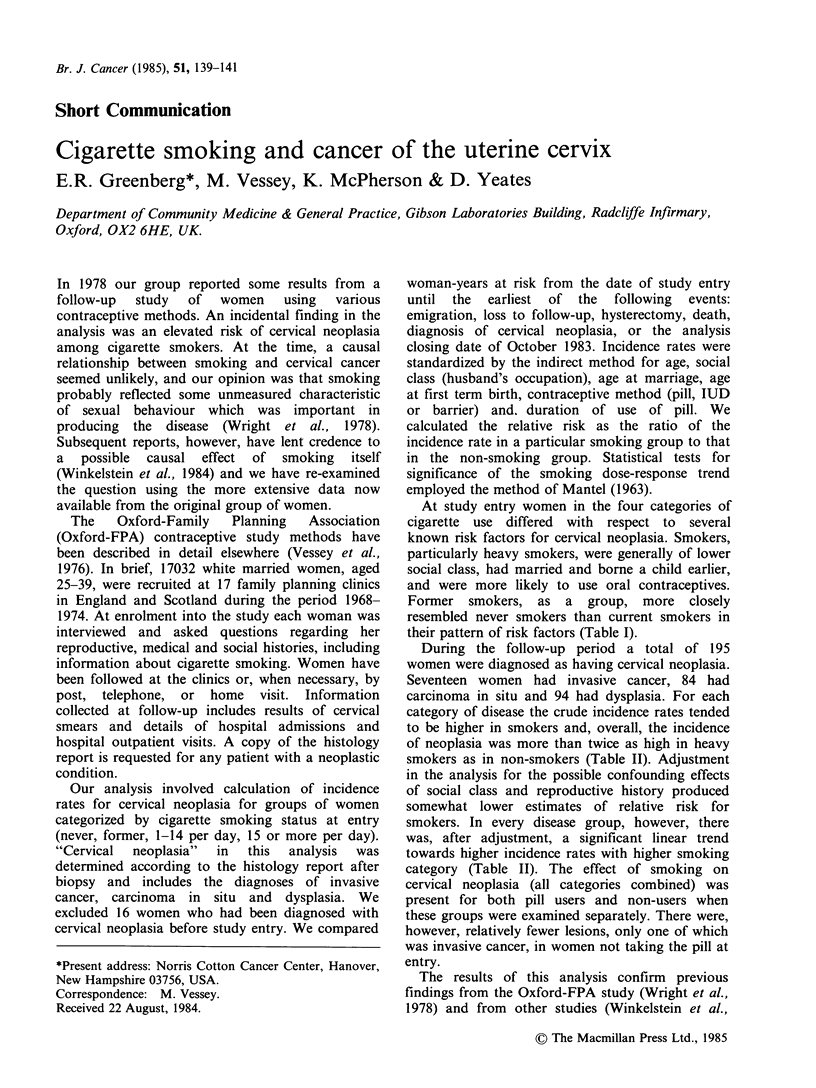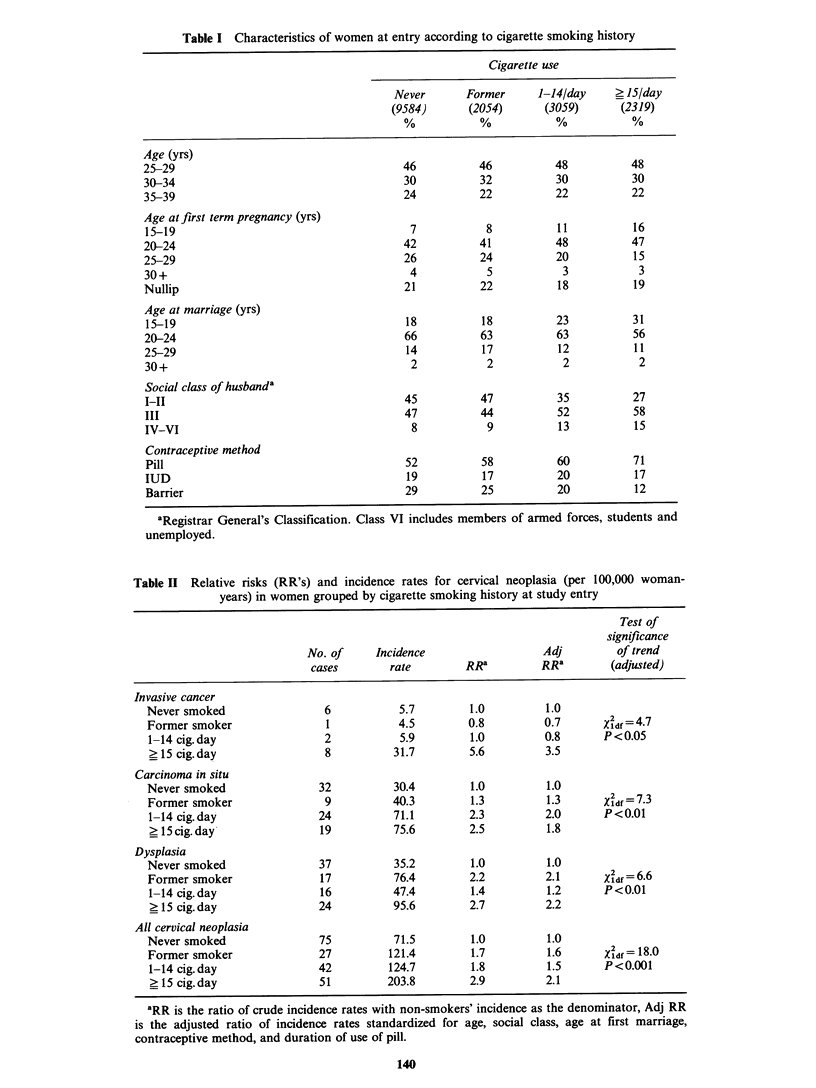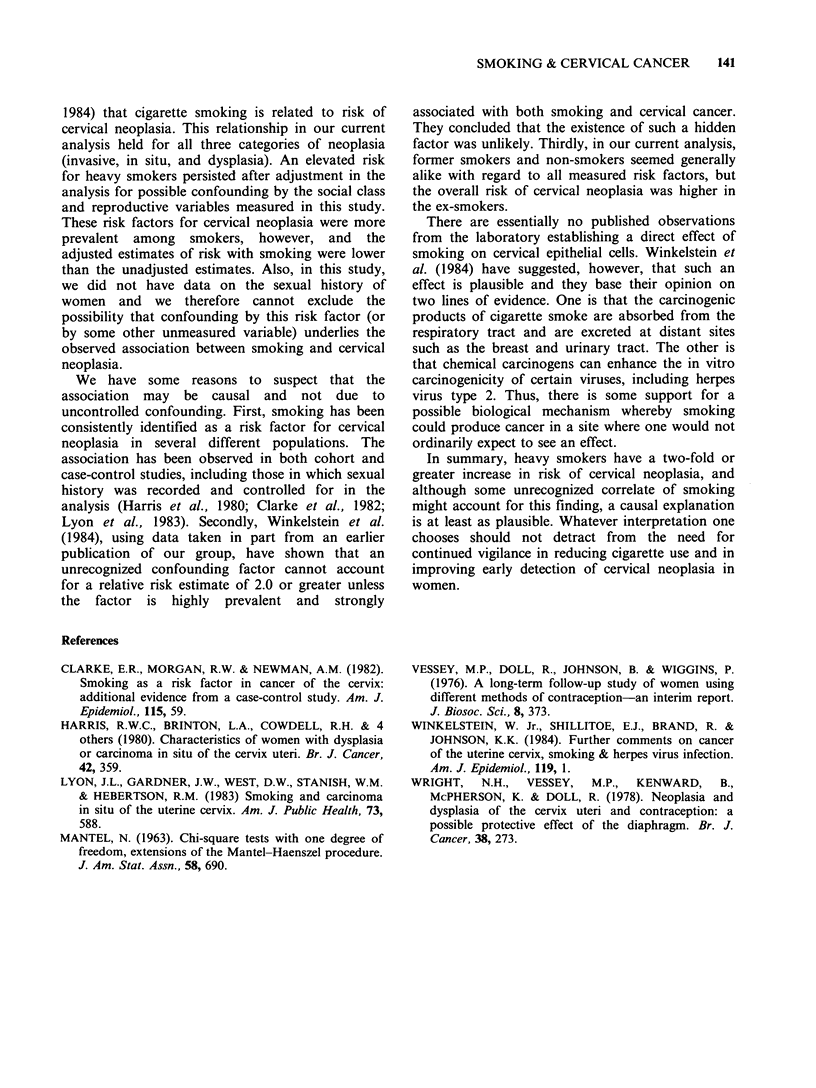# Cigarette smoking and cancer of the uterine cervix.

**DOI:** 10.1038/bjc.1985.21

**Published:** 1985-01

**Authors:** E. R. Greenberg, M. Vessey, K. McPherson, D. Yeates


*Br. J. Cancer* (1985), **51**, 139–141

### Short Communication

### Cigarette smoking and cancer of the uterine cervix

E.R. Greenberg*, M. Vessey, K. McPherson & D. Yeates

*Department of Community Medicine & General Practice, Gibson Laboratories Building, Radcliffe Infirmary, Oxford, OX2 6HE, UK.*

In 1978 our group reported some results from a follow-up study of women using various contraceptive methods. An incidental finding in the analysis was an elevated risk of cervical neoplasia among cigarette smokers. At the time, a causal relationship between smoking and cervical cancer seemed unlikely, and our opinion was that smoking probably reflected some unmeasured characteristic of sexual behaviour which was important in producing the disease (Wright *et al.*, 1978). Subsequent reports, however, have lent credence to a possible causal effect of smoking itself (Winkelstein *et al.*, 1984) and we have re-examined the question using the more extensive data now available from the original group of women.

The Oxford-Family Planning Association (Oxford-FPA) contraceptive study methods have been described in detail elsewhere (Vessey *et al.*, 1976). In brief, 17032 white married women, aged 25–39, were recruited at 17 family planning clinics in England and Scotland during the period 1968–1974. At enrolment into the study each woman was interviewed and asked questions regarding her reproductive, medical and social histories, including information about cigarette smoking. Women have been followed at the clinics or, when necessary, by post, telephone, or home visit. Information collected at follow-up includes results of cervical smears and details of hospital admissions and hospital outpatient visits. A copy of the histology report is requested for any patient with a neoplastic condition.

Our analysis involved calculation of incidence rates for cervical neoplasia for groups of women categorized by cigarette smoking status at entry (never, former, 1–14 per day, 15 or more per day). "Cervical neoplasia" in this analysis was determined according to the histology report after biopsy and includes the diagnoses of invasive cancer, carcinoma in situ and dysplasia. We excluded 16 women who had been diagnosed with cervical neoplasia before study entry. We compared woman-years at risk from the date of study entry until the earliest of the following events: emigration, loss to follow-up, hysterectomy, death, diagnosis of cervical neoplasia, or the analysis closing date of October 1983. Incidence rates were standardized by the indirect method for age, social class (husband's occupation), age at marriage, age at first term birth, contraceptive method (pill, IUD or barrier) and. duration of use of pill. We calculated the relative risk as the ratio of the incidence rate in a particular smoking group to that in the non-smoking group. Statistical tests for significance of the smoking dose-response trend employed the method of Mantel (1963).

At study entry women in the four categories of cigarette use differed with respect to several known risk factors for cervical neoplasia. Smokers, particularly heavy smokers, were generally of lower social class, had married and borne a child earlier, and were more likely to use oral contraceptives. Former smokers, as a group, more closely resembled never smokers than current smokers in their pattern of risk factors (Table I).

During the follow-up period a total of 195 women were diagnosed as having cervical neoplasia. Seventeen women had invasive cancer, 84 had carcinoma in situ and 94 had dysplasia. For each category of disease the crude incidence rates tended to be higher in smokers and, overall, the incidence of neoplasia was more than twice as high in heavy smokers as in non-smokers (Table II). Adjustment in the analysis for the possible confounding effects of social class and reproductive history produced somewhat lower estimates of relative risk for smokers. In every disease group, however, there was, after adjustment, a significant linear trend towards higher incidence rates with higher smoking category (Table II). The effect of smoking on cervical neoplasia (all categories combined) was present for both pill users and non-users when these groups were examined separately. There were, however, relatively fewer lesions, only one of which was invasive cancer, in women not taking the pill at entry.

The results of this analysis confirm previous findings from the Oxford-FPA study (Wright *et al.*, 1978) and from other studies (Winkelstein *et al.*,

*Present address: Norris Cotton Cancer Center, Hanover, New Hampshire 03756, USA.
Correspondence: M. Vessey.
Received 22 August, 1984.

© The Macmillan Press Ltd., 1985

**Table I** Characteristics of women at entry according to cigarette smoking history

| | *Cigarette use* | | | |
|---|---|---|---|---|
| | *Never* (*9584*) % | *Former* (*2054*) % | *1–14/day* (*3059*) % | *≧15/day* (*2319*) % |
| *Age* (yrs) | | | | |
| 25–29 | 46 | 46 | 48 | 48 |
| 30–34 | 30 | 32 | 30 | 30 |
| 35–39 | 24 | 22 | 22 | 22 |
| *Age at first term pregnancy* (yrs) | | | | |
| 15–19 | 7 | 8 | 11 | 16 |
| 20–24 | 42 | 41 | 48 | 47 |
| 25–29 | 26 | 24 | 20 | 15 |
| 30+ | 4 | 5 | 3 | 3 |
| Nullip | 21 | 22 | 18 | 19 |
| *Age at marriage* (yrs) | | | | |
| 15–19 | 18 | 18 | 23 | 31 |
| 20–24 | 66 | 63 | 63 | 56 |
| 25–29 | 14 | 17 | 12 | 11 |
| 30+ | 2 | 2 | 2 | 2 |
| *Social class of husband*[a] | | | | |
| I–II | 45 | 47 | 35 | 27 |
| III | 47 | 44 | 52 | 58 |
| IV–VI | 8 | 9 | 13 | 15 |
| *Contraceptive method* | | | | |
| Pill | 52 | 58 | 60 | 71 |
| IUD | 19 | 17 | 20 | 17 |
| Barrier | 29 | 25 | 20 | 12 |

[a]Registrar General's Classification. Class VI includes members of armed forces, students and unemployed.

**Table II** Relative risks (RR's) and incidence rates for cervical neoplasia (per 100,000 woman-years) in women grouped by cigarette smoking history at study entry

| | *No. of cases* | *Incidence rate* | *RR*[a] | *Adj RR*[a] | *Test of significance of trend (adjusted)* |
|---|---|---|---|---|---|
| *Invasive cancer* | | | | | |
| Never smoked | 6 | 5.7 | 1.0 | 1.0 | |
| Former smoker | 1 | 4.5 | 0.8 | 0.7 | $\chi^2_{1df}=4.7$ |
| 1–14 cig. day | 2 | 5.9 | 1.0 | 0.8 | $P<0.05$ |
| ≧15 cig. day | 8 | 31.7 | 5.6 | 3.5 | |
| *Carcinoma in situ* | | | | | |
| Never smoked | 32 | 30.4 | 1.0 | 1.0 | |
| Former smoker | 9 | 40.3 | 1.3 | 1.3 | $\chi^2_{1df}=7.3$ |
| 1–14 cig. day | 24 | 71.1 | 2.3 | 2.0 | $P<0.01$ |
| ≧15 cig. day | 19 | 75.6 | 2.5 | 1.8 | |
| *Dysplasia* | | | | | |
| Never smoked | 37 | 35.2 | 1.0 | 1.0 | |
| Former smoker | 17 | 76.4 | 2.2 | 2.1 | $\chi^2_{1df}=6.6$ |
| 1–14 cig. day | 16 | 47.4 | 1.4 | 1.2 | $P<0.01$ |
| ≧15 cig. day | 24 | 95.6 | 2.7 | 2.2 | |
| *All cervical neoplasia* | | | | | |
| Never smoked | 75 | 71.5 | 1.0 | 1.0 | |
| Former smoker | 27 | 121.4 | 1.7 | 1.6 | $\chi^2_{1df}=18.0$ |
| 1–14 cig. day | 42 | 124.7 | 1.8 | 1.5 | $P<0.001$ |
| ≧15 cig. day | 51 | 203.8 | 2.9 | 2.1 | |

[a]RR is the ratio of crude incidence rates with non-smokers' incidence as the denominator, Adj RR is the adjusted ratio of incidence rates standardized for age, social class, age at first marriage, contraceptive method, and duration of use of pill.

1984) that cigarette smoking is related to risk of cervical neoplasia. This relationship in our current analysis held for all three categories of neoplasia (invasive, in situ, and dysplasia). An elevated risk for heavy smokers persisted after adjustment in the analysis for possible confounding by the social class and reproductive variables measured in this study. These risk factors for cervical neoplasia were more prevalent among smokers, however, and the adjusted estimates of risk with smoking were lower than the unadjusted estimates. Also, in this study, we did not have data on the sexual history of women and we therefore cannot exclude the possibility that confounding by this risk factor (or by some other unmeasured variable) underlies the observed association between smoking and cervical neoplasia.

We have some reasons to suspect that the association may be causal and not due to uncontrolled confounding. First, smoking has been consistently identified as a risk factor for cervical neoplasia in several different populations. The association has been observed in both cohort and case-control studies, including those in which sexual history was recorded and controlled for in the analysis (Harris *et al.*, 1980; Clarke *et al.*, 1982; Lyon *et al.*, 1983). Secondly, Winkelstein *et al.* (1984), using data taken in part from an earlier publication of our group, have shown that an unrecognized confounding factor cannot account for a relative risk estimate of 2.0 or greater unless the factor is highly prevalent and strongly associated with both smoking and cervical cancer. They concluded that the existence of such a hidden factor was unlikely. Thirdly, in our current analysis, former smokers and non-smokers seemed generally alike with regard to all measured risk factors, but the overall risk of cervical neoplasia was higher in the ex-smokers.

There are essentially no published observations from the laboratory establishing a direct effect of smoking on cervical epithelial cells. Winkelstein *et al.* (1984) have suggested, however, that such an effect is plausible and they base their opinion on two lines of evidence. One is that the carcinogenic products of cigarette smoke are absorbed from the respiratory tract and are excreted at distant sites such as the breast and urinary tract. The other is that chemical carcinogens can enhance the in vitro carcinogenicity of certain viruses, including herpes virus type 2. Thus, there is some support for a possible biological mechanism whereby smoking could produce cancer in a site where one would not ordinarily expect to see an effect.

In summary, heavy smokers have a two-fold or greater increase in risk of cervical neoplasia, and although some unrecognized correlate of smoking might account for this finding, a causal explanation is at least as plausible. Whatever interpretation one chooses should not detract from the need for continued vigilance in reducing cigarette use and in improving early detection of cervical neoplasia in women.

### References

CLARKE, E.R., MORGAN, R.W. & NEWMAN, A.M. (1982). Smoking as a risk factor in cancer of the cervix: additional evidence from a case-control study. *Am. J. Epidemiol.*, **115**, 59.

HARRIS, R.W.C., BRINTON, L.A., COWDELL, R.H. & 4 others (1980). Characteristics of women with dysplasia or carcinoma in situ of the cervix uteri. *Br. J. Cancer*, **42**, 359.

LYON, J.L., GARDNER, J.W., WEST, D.W., STANISH, W.M. & HEBERTSON, R.M. (1983) Smoking and carcinoma in situ of the uterine cervix. *Am. J. Public Health*, **73**, 588.

MANTEL, N. (1963). Chi-square tests with one degree of freedom, extensions of the Mantel–Haenszel procedure. *J. Am. Stat. Assn.*, **58**, 690.

VESSEY, M.P., DOLL, R., JOHNSON, B. & WIGGINS, P. (1976). A long-term follow-up study of women using different methods of contraception—an interim report. *J. Biosoc. Sci.*, **8**, 373.

WINKELSTEIN, W. Jr., SHILLITOE, E.J., BRAND, R. & JOHNSON, K.K. (1984). Further comments on cancer of the uterine cervix, smoking & herpes virus infection. *Am. J. Epidemiol.*, **119**, 1.

WRIGHT, N.H., VESSEY, M.P., KENWARD, B., McPHERSON, K. & DOLL, R. (1978). Neoplasia and dysplasia of the cervix uteri and contraception: a possible protective effect of the diaphragm. *Br. J. Cancer*, **38**, 273.